# Aberrant Cell Cycle and Apoptotic Changes Characterise Severe Influenza A Infection – A Meta-Analysis of Genomic Signatures in Circulating Leukocytes

**DOI:** 10.1371/journal.pone.0017186

**Published:** 2011-03-08

**Authors:** Grant Parnell, Anthony McLean, David Booth, Stephen Huang, Marek Nalos, Benjamin Tang

**Affiliations:** 1 Department of Intensive Care Medicine, Western Clinical School, Nepean Hospital, University of Sydney, New South Wales, Australia; 2 Westmead Millennium Institute, University of Sydney, New South Wales, Australia; The University of Hong Kong, Hong Kong

## Abstract

Influenza A infection is a global disease that has been responsible for four pandemics over the last one hundred years. However, it remains poorly understood as to why some infected individuals succumb to life threatening complications whilst others recover and are relatively unaffected. Using gene-expression analysis of circulating leukocytes, here we show that the progression towards severe influenza A infection is characterised by an abnormal transcriptional reprogramming of cell cycle and apoptosis pathways. In severely infected humans, leukocyte gene-expression profiles display opposing cell cycle activities; an increased aberrant DNA replication in the G_1_/S phase yet delayed progression in the G_2_/M phase. In mild infection, cell cycle perturbations are fewer and are integrated with an efficient apoptotic program. Importantly, the loss of integration between cell cycle perturbations and apoptosis marks the transition from a mild viral illness to a severe, life threatening infection. Our findings suggest that circulating immune cells may play a significant role in the evolution of the host response. Further study may reveal alternative host response factors previously unrecognized in the current disease model of influenza.

## Introduction

Disease progression remains poorly understood in influenza A infection. Each year, millions of individuals worldwide are infected with influenza virus [1]. It remains unknown as to why some became critically ill whilst others infected with the same virus remain relatively unaffected. Although vvirus related factors have been proposed as influencing disease progression, data from recent pandemic H1N1 2009 influenza shows that the similar viral loads were found in the infected hosts regardless of disease severity [2], [3]. Host response has also been suggested to play a role. However, its exact contribution to disease progression has been for a long time a matter of debate. While some studies show that an exaggerated inflammatory response may be responsible [4], [5], others have shown that a delayed/reduced inflammatory response can also contribute [6].

A better understanding of how host response determines the progression of influenza infection is critically important for two reasons. First, a greater insight into the mechanisms that modulate host response may lead to the development of new therapeutic agents. Second, clinical manifestation of influenza infection is highly variable making it difficult to identify at-risk individuals. Discovering new markers that indicate a decompensated host response will assist clinicians in identifying individuals who are more likely to progress to a more severe infection. Such a risk stratification approach will allow clinicians to deliver prompt treatment to at-risk individuals and hence reduce the fatality rate from influenza-related complications.

Current understanding of influenza infection is limited by the lack of an appropriate human model. Data supporting the established model of influenza infection are predominantly from *in vitro* and animal studies [7]. The pathophysiology of these models, however, may profoundly differ from that in humans. Here, we report the first human model that examines the role of host response in influencing disease progression in influenza A infection. Using gene-expression data derived from circulating leukocytes in infected humans, we examined influenza induced changes in signalling and metabolic maps covering the full spectrum of known molecular pathways in human biology. We show that dysregulated cell cycle activities in circulating leukocytes characterise the progression to severe infection. We also demonstrate that the loss of a coupling relationship between cell cycle perturbation and apoptotic response in circulating leukocytes marks the difference between a well contained, uncomplicated viral illness and a rapidly progressing, severe infection. Put together, these data implicate a major role of circulating leukocytes in influencing disease outcomes in influenza infection.

## Results

To identify the unique pathways that characterized progression from mild to severe illness, we performed a meta-analysis of five microarray data sets. This analysis compared pathway data between different categories of human influenza virus infection, with each category representing a different stage of immune activation (Fig. S1). These categories included (1) healthy subjects after influenza vaccination (hereafter referred to as “Post-Vaccination” group), (2) asymptomatic subjects with influenza A H3N2 infection (hereafter referred to as “Asymptomatic” group), (3) symptomatic subjects with influenza A H3N2 infection (hereafter referred to as “Symptomatic” group) and (4) critically ill subjects with influenza A H1N1 pneumonia (hereafter referred to as “Severe” group). An additional group of critically ill subjects with bacterial pneumonia was included as the positive control (hereafter referred to as “Bacterial” group). The use of positive control allowed us to distinguish between a generic host response (found in most infection, whether it is viral or bacterial) and a specific host response attributable due to influenza viral infection. A total of 55 subjects were included in the analysis. The demographic and clinical information of the included subjects are given in table 1. Immunocompromised patients (e.g., history of receiving corticosteroids therapy or immunosuppressive medications, transplant recipients, haematological malignancies) were excluded from our study. Hierarchical clustering of global gene expression using centred correlation and average linkage was performed for each of the data sets and shows that samples within each particular group tend to cluster together (Fig. S2).

**Table 1:** Patient characteristics in the included studies.

	No. of Subjects	AgeMean (range)	GenderFemale/Male	APACHEII score - mean (range)	Site of infection	Survival/Death	Length of follow-up
Severe influenza infection	4	33 (21–48)	3/1	14 (13,17)	Lung	4/0	5 days
Severe bacterial pneumonia	6	63 (52–75)	3/3	22 (10,33)	Lung	4/2	5 days
Mild influenza infection	9	NA	NA	NA	Lung	9/0	3.5 days
Post-vaccination subjects	18	43 (24–70)	12/6	NA	NA	18/0	7 days
Healthy controls	18	43 (24–70)	12/6	NA	NA	18/0	1 day

APACHE denotes Acute Physiology and Chronic Health Evaluation II scores. NA denotes not available or not applicable.

**Figure 1:**
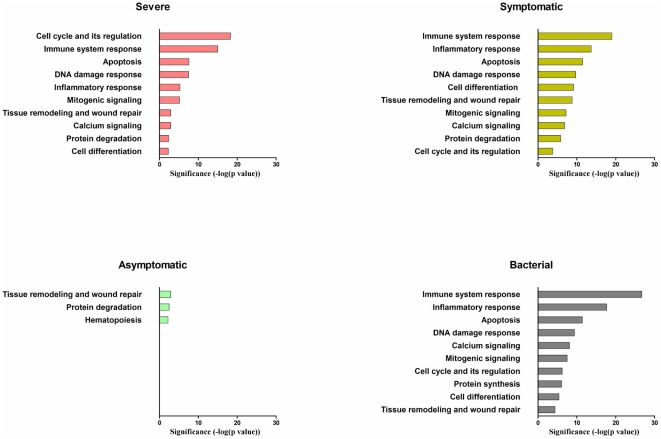
Top significant biological processes during host response to influenza. P-value distribution of the most significant biological processes during host response to influenza infection in Severe, Symptomatic and Asymptomatic groups; Post-Vaccination group is not shown as no significant pathway is represented in this group. Bacterial group is included as a control.

**Figure 2:**
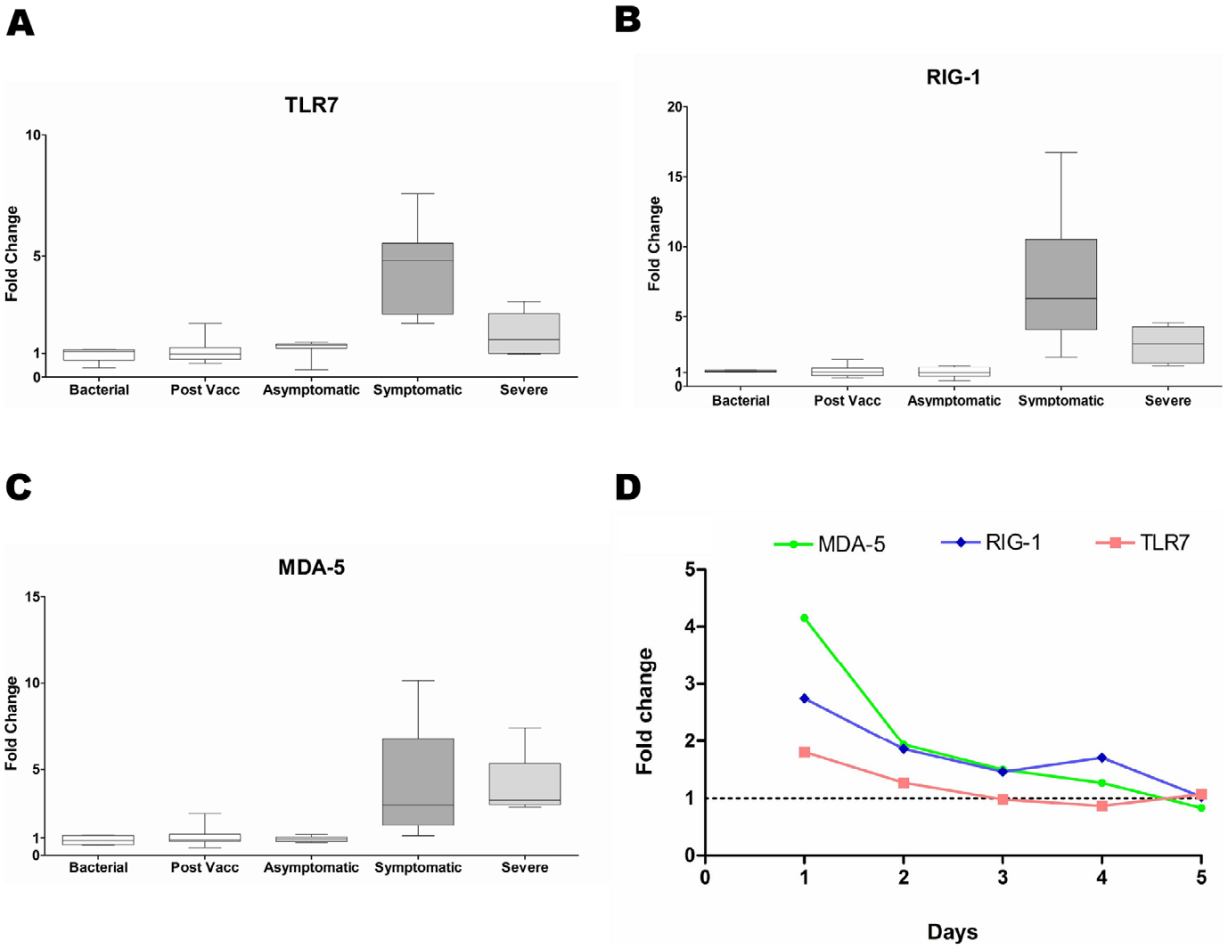
Expression levels of viral detection genes. Expression levels of viral detection genes (**A**) TLR-7, (**B**) RIG-1 and (**C**) MDA-5 in all groups. (**D**) Expression level of viral detection genes TLR-7, RIG-1 and MDA-5 as infection resolves. Base-line expression level is represented as fold-change of 1.

We found that infection severity correlates with the extent of systemic host response. An intense systemic response is seen in the Severe and Symptomatic groups (Fig. 1). In contrast, a minimal response is seen in the Asymptomatic group and none at all in the Post-vaccination group. Activation of this host response correlates with the expression of the virus detection genes *TLR7* (Toll-like receptor 7), *RIG-1* and *MDA-5*. In the Severe and Symptomatic groups, these genes are highly expressed whereas in the Post-vaccination or Asymptomatic groups, there is minimal expression of these genes (Fig. 2A, 2B, 2C). In the Symptomatic and Severe groups, the activation signal is seen in both external and internal viral recognition systems. *TLR7*, the receptor for detecting virus antigens on the host cell surface, shows up to a five-fold increase in gene-expression. *RIG-1* and *MDA-5*, the intra-cellular alarm system for detecting viral RNA, show up to a six-fold increase. There is evidence of viral-induced apoptosis, which is consistent with the increase in expression of *TLR-7, RIG-1* and *MDA-5*. The PKR-dependent apoptosis pathway, known to be involved in influenza virus infection, is activated in both the Symptomatic and Severe groups (Fig. S3A, S3B). There is also a concurrent activation of the anti-viral pathway mediated by type I interferon genes, with up to a ten-fold increase in some of these genes (Fig. S4A). As infection resolves, the viral detection signal declines and this is followed by the return of the interferon response to a quiescent state (Fig. 2D, Fig. S4C).

We found that the systemic host response in severe infection differs significantly from that of mild infection. The main differences lay in the cell cycle and apoptosis pathways. Unexpectedly, immune response pathways did not differ significantly between infected groups. Other than *TNF* and *IL-beta*, inflammation-related genes that are well established in influenza infection do not discriminate between these groups (Fig. S4B). Also, interferon response genes do not differ significantly between mild and severe influenza infection (Fig. S4A). The lack of correlation among established immune/inflammatory markers led us to postulate that disease progression is determined by changes occurring elsewhere, such as in the cell cycle and apoptosis pathways.

**Figure 3:**
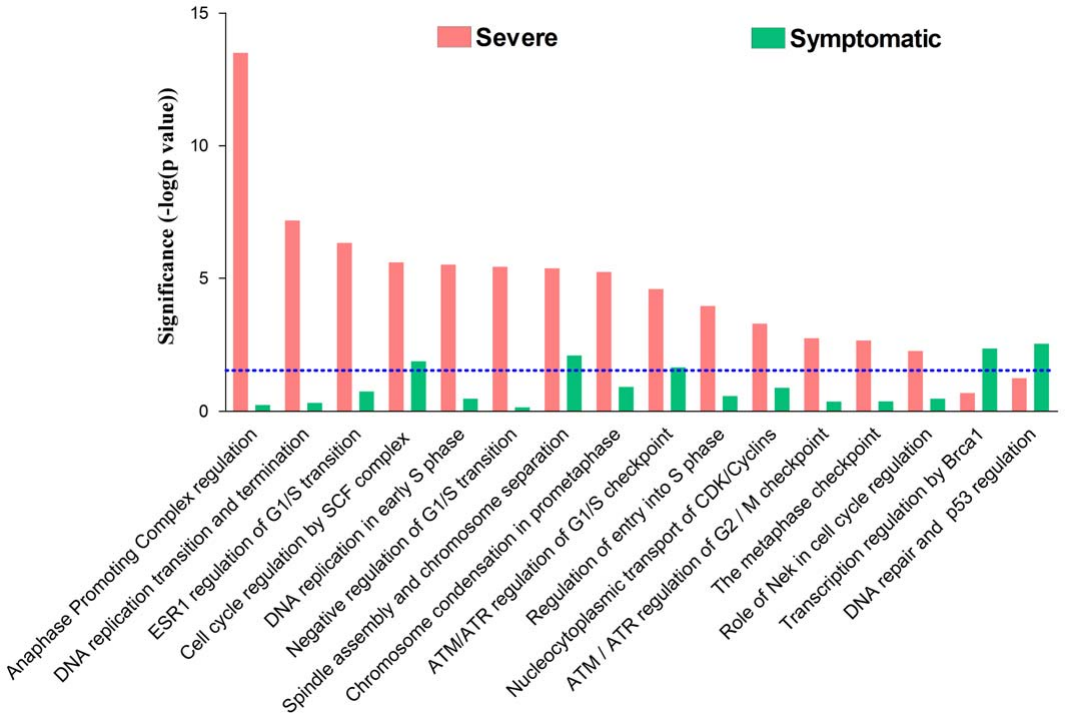
Cell cycle perturbations during influenza A infection. Cell cycle pathways in severe and mild influenza infection, represented here by Severe and Symptomatic groups.

**Figure 4:**
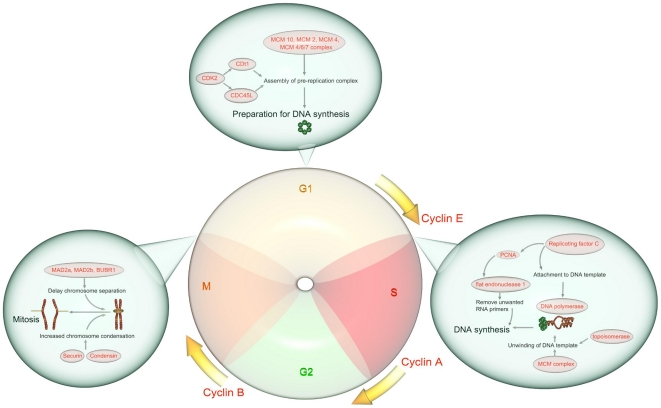
Cell cycle genes in severe influenza infection. Only statistically significant genes are shown. Cell cycle phases are represented as G_1_, S, G_2_ and M. Up-regulated genes are coloured red and enclosed in ovals. Cyclin A, B and E are also up-regulated.

Further analyses revealed that there is a significantly greater number of cell cycle pathways activated in severe influenza infection compared to mild infection (Fig. 3). In addition, the Severe group shows a greater up-regulation of genes encoding for key cell cycle proteins (Fig. 4). These cell cycle proteins include cyclin and their associated catalytic kinase enzymes, namely, cyclin E (G_1_–S phase transition), cyclin A (S-phase progression), cyclin B (G_2_–M phase transition), CDK1 and CDK2. Furthermore, this up-regulation is accompanied by an extensive activation of DNA replication machinery, including the pre-replication complex assembly, MCM complex and Cdt1 (Fig. S5A, S5B). The heightened DNA replication activity does not seem to be host cell initiated because cyclin D, the initiator of cell cycle, is paradoxically down-regulated. Importantly, the increased DNA synthesis occurs in the context of an abnormally low leukocyte response to infection (Fig. S5E), indicating that it is not a physiologically normal response.

Despite an increase in DNA synthesis, paradoxical changes were seen in the mitotic phase. Here, we found up-regulation of genes opposing the completion of mitosis (Fig. 4), including those encoding Securins (inhibitor of chromosomes separation) and the Condensin Complex (structural maintenance of chromosomes). Furthermore, there is strong activation of the spindle checkpoint complex (MD2a, MD2b and BUBR1), the cellular sensing system that normally prevents premature separation of chromosomes. Together, these proteins maintain chromosome condensation and their up-regulation is known to be associated with delayed mitotic exit [8]. To understand the mechanism underlying this finding, we focused on the anaphase promoting complex (APC), the major regulatory complex that coordinates cell cycle progression and exit from mitosis [9], which was also the most statistically significant pathway found in our analysis (Fig. 3). Here we found abnormal changes in APC and its two co-activators (CDC20 and hCDH1). In subjects with a severe infection, CDC20 is unusually upregulated whilst no activation is seen in hCDH1 (Fig. S5C). Most importantly, the APC gene is not expressed at all. In summary, severe influenza infection is characterized by opposing changes in cell cycle activity (accelerating DNA synthesis but delayed mitotic exit) and these changes are associated with dysregulated cell cycle control.

**Figure 5:**
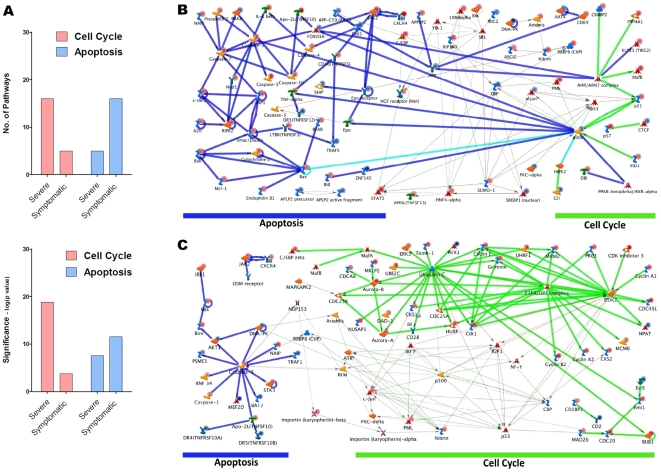
Relationship between apoptosis and cell cycle. (**A**) Apoptosis and cell cycle pathways during influenza infection. Direct interaction networks for cell cycle and apoptosis genes in (**B**) mild influenza A infection and (**C**) severe influenza A infection. Dark blue lines represent apoptosis whereas green lines represent cell cycle pathways. The pale blue line indicates that these genes are involved in both apoptosis and cell cycle pathways. The thin edges represent the expanded network of the DNA-damage response pathway. Coloured circles above individual genes indicate up (red) or down (blue) regulation.

In contrast to changes in cell cycle, the apoptosis pathways were activated to a greater degree in mild infection than in severe infection (Fig. 5A). Given that cell cycle perturbations are known to trigger apoptosis [10], we proceeded to investigate if host cell related mechanisms (via cell cycle genes) may be implicated in causing this difference. Nibrin, GADD45 and PCNA, which are cell cycle genes involved in detecting genetic damage and promoting DNA repair, are highly expressed in both the Severe and Symptomatic groups (Fig. S5D). Importantly, the genes which link DNA-damage response to apoptosis are also up-regulated. We therefore used network analysis to further explore the relationship between cell cycle and apoptosis genes. We first built networks (by direct interaction) using apoptosis and cell cycle genes separately. Within the cell cycle network, connectivity for DNA-damage response genes was further expanded. Cell cycle and apoptosis networks were then merged so that we could identify any reciprocal relationship between these networks. This analysis revealed that, in mild infection, the cell cycle network is highly integrated with an efficient programmed cell death response (Fig. 5B). The integration is mediated predominantly via a p53-dependent DNA-damage response pathway. In contrast, such integration is lost in severe infection. Here, the DNA-damage response signals are not only considerably weaker, but they also fail to couple with the apoptosis network (Fig. 5C). This may reflect the host's attempt, albeit unsuccessful, to limit genome damage and restore homeostasis during influenza infection. Since apoptosis allows the host to eliminate non-viable cells and limit virus replication, the loss of this self-preservation response, combined with cell cycle perturbations, may mark the difference between mild and severe infection.

**Figure 6:**
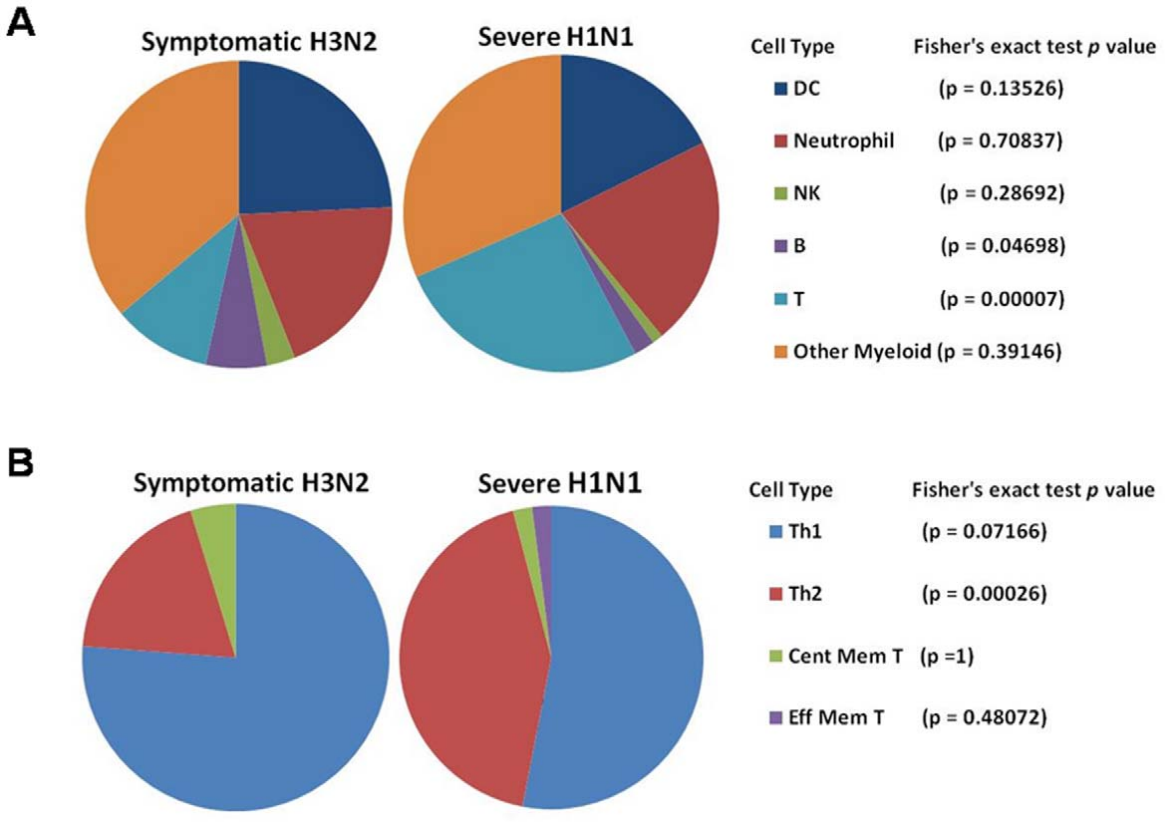
Cell tagging via ImmGen – Symptomatic and Severe influenza infection. Representation of (**A**) 6 immune cell subsets and (**B**) T cell subsets in the top 100 up-regulated genes of the Symptomatic and Severe influenza infection groups.

The above observations also reveal important differences between severe and mild infection. In severe infection, host circulating leukocytes undergo extensive transcriptional reprogramming (increased G_1_/S phase activity, delayed G_2_/M phase progression and de-coupling of the cell cycle-apoptosis relationship). In contrast, mild infection shows considerably fewer changes and asymptomatic infection shows no changes at all. These differences may reflect shifting changes in immune cell populations during different stages of the influenza infection. For example, in the early stage of influenza infection, CD4^+^ cells differentiate into T-helper 1 cells (Th1) and a bias towards Th1 cells development protects host from severe infection [11]. We therefore examined the cellular origins of the gene-expression signals detected in both severe and mild influenza infection, in order to see if the changes in immune cell populations mirror the difference in transcriptional profiles between these two groups (Fig. 6). Using cell tagging via the ImmGene, we found that gene-expression signals related to Th2 development are more represented in the severe influenza group compared to the mild influenza group (p = 0.00026). On the other hand, Th1 cells are more represented in the mild influenza group (p = 0.072). This finding confirms that the observed differences in transcriptional activities do parallel a shift in immune cell sub-populations between mild and severe infection. However, the precise relationship between transcriptional reprogramming and specific immune cells sub-populations remains unknown and this warrants further study.

## Discussion

A prevailing view in the established model of influenza infection is that dysregulated inflammatory response in the lungs drives the progression towards a more severe disease [4], [5], [6], [12]. However, the evidence for this mechanism is based almost exclusively on animal studies [7]. Emerging human data from studies of the recent H1N1 virus pandemic revealed that host factors beyond that of lung parenchyma are also likely to contribute to disease progression [2]. We therefore developed a human model of influenza A infection to delineate these systemic host factors and to understand their role in driving disease progression in infected individuals. Using a human model incorporating varying infection severity, we found important differences in pathway profiles of circulating leukocytes between asymptomatic, mild and severe influenza infection. The severe influenza infection group shows the most profound changes by having the greatest number of cell cycle pathways perturbed. These changes are characterized by an increased aberrant DNA replication in the G_1_/S phase but a delayed exit from the G_2_/M phase. The increased DNA replication is associated with an impaired leukocyte response to infection, suggesting that the delayed mitotic exit may be the key step that limits leukocyte proliferation during influenza infection. Given that circulating immune cells constitute the main effector arm of the cell mediated response against influenza viral infection, our findings suggest that these cells may play a major role in determining the outcome of influenza infection.

Our findings on cell cycle perturbations are consistent with emerging experimental data. Aberrant DNA replication activity has recently been shown to occur during influenza virus infection [13]. The MCM complex, a helicase involved in eukaryotic DNA replication, has been identified as the host factor used by influenza A virus to increase viral replication [13]. Delayed mitotic exit has also been implicated in the pathogenesis of viral infection and it is thought to be caused by dysregulation of the APC [8]. In our study, both up-regulation of the MCM complex and dysregulation of the APC are evident in the most severely infected patients.

Our findings also reveal a critical role of apoptosis in influenza infection. While apoptosis has been widely reported in studies of influenza infection, its implication on disease progression has not been well understood. Conflicting evidence exist as to whether apoptosis is harmful or beneficial to the host during influenza infection [14]. Our findings demonstrate that, rather than apoptosis per se, it is the coupling relationship between cell cycle perturbations and apoptosis that may influence the outcome of the disease. Furthermore, our data suggests that this coupling relationship is mediated via the p53-dependent pathway, a well established self-repair pathway that limits DNA damage and cell cycle perturbations in host cells. Recent evidence supports this finding. In influenza virus infected human lung cells, p53 is shown to be essential for the induction of apoptosis and its inhibition resulted in elevated virus replication [15]. In mice infected with the influenza virus, an increased activation of the p53 dependent DNA-damage response (G_2_/M checkpoint) is associated with reduced lung inflammation and better survival [5].

**Figure 7:**
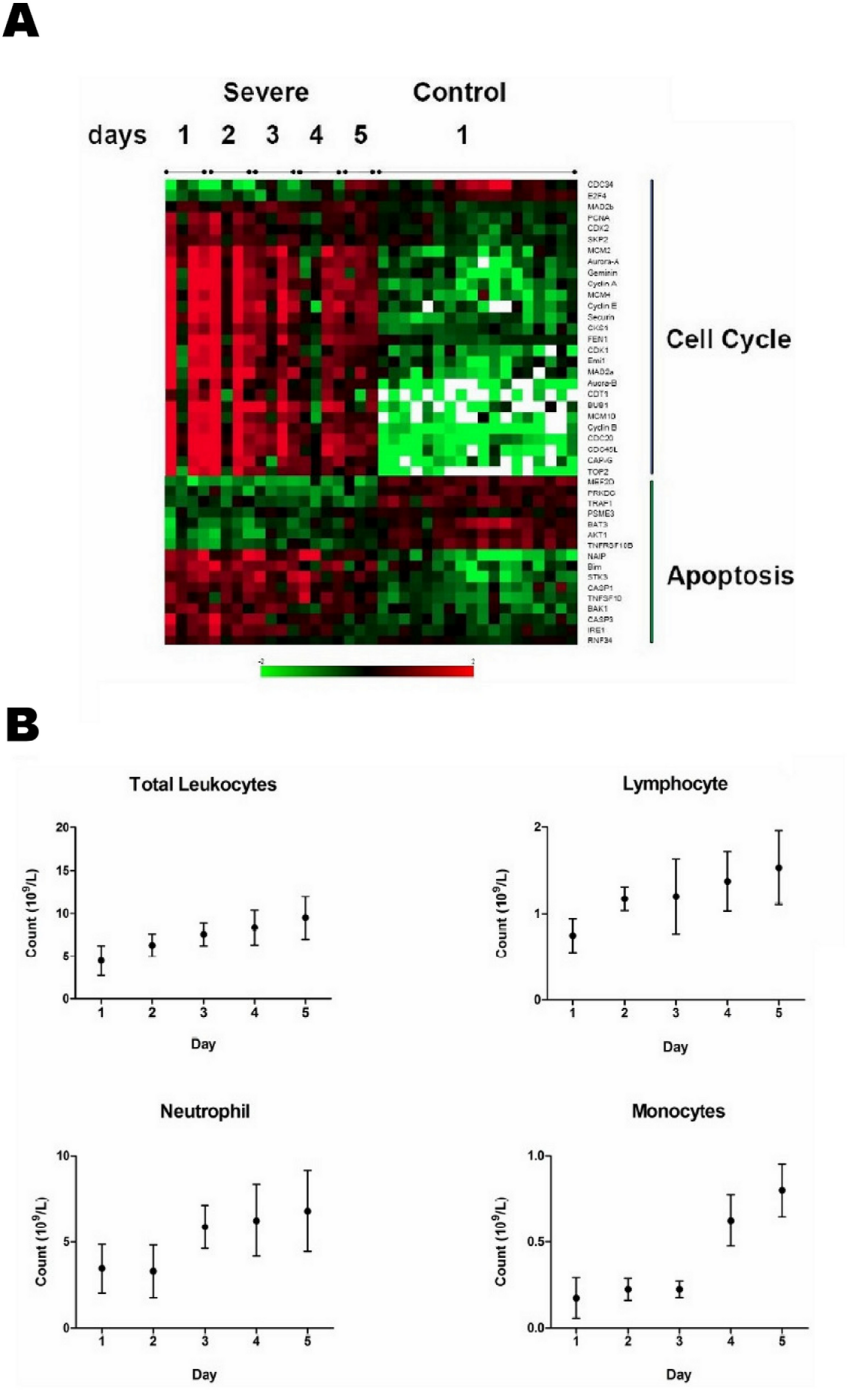
Recovery from severe influenza A Infection. (**A**) Attenuation of apoptosis and cell cycle expression levels during recovery. Control refers to healthy volunteers. (**B**) Recovery of total leukocytes, lymphocytes, monocytes and neutrophils as infection resolves in the Severe group. Immune cell counts were collected as part of the routine clinical tests performed in the ICU.

Put together, our findings reveal a systematic loss of control by the host leukocytes over key cellular functions, including DNA synthesis, mitotic exit and self-repair response. As infection resolved, these perturbations subsided and were accompanied by a recovery in host response including lymphocyte, monocyte and neutrophil cell counts (Fig. 7a, 7b). Leukocyte proliferation is an important of part of the host immune response and is critical for the clearance of influenza infection [16]. Cell cycle perturbations may impair leukocyte proliferation, leading to a significantly diminished host response and consequently a more severe infection. Our results therefore suggest a plausible mechanism for explaining why some individuals succumb to severe influenza infection whilst others recover quickly after having only a relatively mild illness.

Previous studies have focused predominantly on the role of immune cells in lung parenchyma and local lymphoid tissue in driving disease progression. It is commonly believed that disease progression occurs when influenza virus replication overwhelms the local defence mediated by immune cells in the lung. However, such a model fails to explain why viral loads assayed from airway samples in infected human are similar regardless of disease severity, a finding consistently observed in the recent pandemic H1N1 influenza infection [3], [17], [18]. This study provides an alternative mechanism to explain disease progression during influenza infection. Our findings suggest that, during primary influenza infection, an unabated cell cycle dysregulation in systemic immune cells diminishes host response. Host cells respond by programmed cell death to eliminate non-viable cells and to limit genome damage. The manner by which the influenza virus modulates this response may very well represent the distinguishing features between a mild, self-limiting illness and a highly lethal infection.

There are limitations in our study. First, different influenza virus strains were used in the study. Ideally, each group being infected with the same virus will allow a more valid comparison between the groups. Second, an inherent limitation with observational study is that groups differ not just in the phenotype of interest (severe infection vs. mild infection), but also in other characteristics as well (e.g. age, gender, co-existing medical conditions). These other characteristics could have confounded our findings. Third, two different microarray platforms were used in generating data for this study. The difference in technology may have introduced artifectual differences that may potentially dilute the real biological signals.

In conclusion, our study extends the established influenza model to include a key role for circulating leukocyte response to infection. The discovery of a significant pathology in circulating leukocytes provides an additional perspective from which to interrogate the role of host response in influenza infection. It also provides an opportunity to develop new diagnostic and therapeutic approaches. Importantly, the discovery of infection progression signature markers may help address the prevailing challenge in an influenza pandemic, namely, to distinguish high risk individuals from a vast number of uncomplicated, self-limited infected cases. An improved ability to stratify, select and protect high risk individuals has major public health implications, as we anticipate the arrival of the next influenza pandemic.

## Materials and Methods

### Subjects

Critically ill patients with severe infection were enrolled in our study. Severe infection is defined as infection where there is at least one major organ failure (e.g. respiratory failure) that requires critical care intervention (e.g. mechanical ventilation). Viral infection (n = 4) was confirmed using PCR and bacterial infection (n = 6) by microbiological cultures. Healthy volunteers (n = 18) were enrolled from a local influenza vaccination program. Whole blood samples were drawn from all subjects. For critically ill patients, sampling coincided with their peak clinical symptoms. These critically ill patients were followed up for a further four days to assess their recovery profiles. A previously published report provide the gene-expression data of 17 subjects with mild seasonal influenza infection [19]. In symptomatic subjects (n = 9), gene-expression data on the day of peak symptoms was analysed. In asymptomatic subjects (n = 8), gene-expression data obtained after an average of 3.5 days was analysed.

### Viruses

Subjects from the Symptomatic and Asymptomatic groups were infected with the seasonal H3N2 influenza virus. Subjects from the critically ill viral infection group were infected with the pandemic H1N109 influenza virus.

### Expression analysis

Samples were collected into PAXgene tubes. Upon collection, the samples were immediately stored at minus 20 degrees Celsius. RNA extraction was performed in batches of 12–24 samples at a time. Samples were first incubated at room temperature for 3 hours before following the standard RNA extraction protocol for the PAXgene RNA extraction kit (PAXgene^TM^ Blood RNA kit - Qiagen, Germany). Extracted RNA was then stored at minus 80 degrees Celsius until required for amplification and labelling using Illumina TotalPrep Amplification kit. Prior to sample amplification and labelling, RNA quality was analysed using Agilent Bioanalyser and all samples obtained a RIN of greater than 6.5. Amplification and labelling was carried out 24 samples at a time. 200ng of Total RNA was used as the starting quantity for amplification and labelling of all samples. Once the amplification and labelling was completed, the amplified cRNA was also assessed using the Agilent Bioanalyser, to ensure satisfactory amplification. The samples were then immediately hybridised on the HT-12 beadchips. 750ng of each sample was loaded on to the arrays. The hybridisation and washing procedure was identical for each set of arrays processed and after normalisation, no significant batch effects were identified.

All of the RNA extraction, sample amplification and labelling, hybridisation and washing, and scanning procedures were carried out by the same operator, at the same time of day. Sample signals were normalized with cubic spline and then log-transformed prior to analysis. All microarray data are available at GEO (GSE20346), in accordance with minimum information about a microarray experiment (MIAME) standards.

### Bioinformatic workflow

Five data sets were analysed (Fig. S1). Analysis of each data set began with the identification of a signature gene list from each data set. This is done by comparing the diseased patients (e.g. mild influenza infection) to a group of control subjects (healthy volunteers). This generates a list of differentially expressed genes that represents an unique signature for that disease status. Differential expression analysis was performed in each data set using BRB-ArrayTools. In groups with a longitudinal study design, differentially expressed genes were identified using the ANOVA mixed effects model, with disease and time as fixed effects factors and subject as random factor. In groups with a before-and-after study design, differentially expressed genes were identified using the paired t-test (Fig. S1).

When generating differentially expressed genes, the diseased group was compared to the healthy controls within the same cohort. Hence each patient group was compared to its own control group on the same microarray platform (e.g. Affymetrix), ensuring that the comparison between groups was not confounded by the difference in technology (e.g. Affymetrix vs. Illumina).

To undertake pathway analysis, the generated differentially expressed genes were uploaded into the GeneGO™ MetaCore™ (St. Joseph, MI, USA). MetaCore is an integrated software suite for functional analysis of gene-expression data. The software is based on an extensively curated database of protein structures and molecular interactions, and is substantially more comprehensive than the knowledge base provided by KEGG and Biocarta. Using MetaCore, pathway analysis and network analysis were performed in each data set. Pathway analysis involves matching a list of pre-specified genes onto canonical pathways and calculating the statistical relevance of the matches found. Each canonical pathway represents the current consensus knowledge of a specific biological process including intracellular cell signalling, regulatory processes and metabolic processes. Results are presented as pathway maps with overlaying gene-expression data. A false discovery rate of 5% is used as the cut-off to determine if a pathway is statistically significant. Network analysis was performed within GeneGo using pre-specified genes as root objects and then subsequently expanded based on known biological relationships and protein/gene interactions.

### Cell tagging

To identify the cellular sources of the gene-expression signals, we performed cell tagging analysis using ImmGen. ImmGen is a public data gene-expression repository consisting of whole-genome microarray datasets for nearly all characterized cell populations of the adaptive and innate immune systems [20]. Using the query function in the ImmGen, all the immune cell subtypes that express a particular gene can be identified (cell tagging) [21]. This approach allows identification of the multiple cell types that express the same gene, as well as knowing whether the gene is expressed in either the activated state or the resting state of the cell. To identify the immune cell sub-populations that give rise to the most significant genes, the top 100 highest-ranking up-regulated genes from the Symptomatic H3N2 and Severe H1N1 groups were used. Each gene was then searched in ImmGen using the immunological genome browser for human immune cells (e.g. monocytes, dendritic cells, Th1 and Th2). The cell types that express the top 100 significant genes were then collated for both the Symptomatic and the Severe groups. Fisher's exact test is then used to determine whether the representation of any particular immune cell type is statistically different between the two groups.

### PCR

We performed qPCR on selected cell cycle and apoptosis genes. The findings from qPCR show that the gene-expression level correlates well with those from microarray experiments. These findings are presented in Table S1.

### Data Deposition

All microarray data has been deposited in GEO (record number - GSE20346).

## Supporting Information

Figure S1 **Schematic representation of study design and bioinformatic workflow.**
(TIF)

Figure S2 **Hierarchical clustering of global gene expression using average linkage and centred correlation.** (**A**) Heatmap of data sets assayed on the Illumina Platform. (**B**) Heatmap of data sets assayed on the Affymetrix platform.
(TIF)

Figure S3 **PKR-dependent apoptosis in mild (A) and severe (B) influenza A infection.** Pale blue lines indicate direct interaction with PKR. Coloured circles above individual genes indicate up (red) or down (blue) regulation.
(TIF)

Figure S4 **Interferon and Inflammatory response in mild and severe influenza A infection.** (**a**) Expression level of interferon related genes in mild and severe influenza A infection. Day 1 samples are shown for the severe group. (**b**) Expression level of inflammatory cytokine genes. Day 1 samples are shown for the severe group. (**c**) Heatmap of interferon related genes during recovery in severe influenza A infection.
(TIF)

Figure S5 **DNA replication and Cell cycle pathways and leukocyte response in severe influenza A infection.** **(a) Pathway diagram for start of DNA replication in early S phase.** Red bars indicate up-regulation and blue bars indicate down-regulation. Bars labelled 1 refer to severe influenza A infection and bars labelled 2 refer to mild influenza A infection. A detailed description of this map can be found at http://www.genego.com/map_705.php. **(b) Pathway diagram for transition and termination of DNA replication.** Red bars indicate up-regulation and blue bars indicate down-regulation. Bars labelled 1 refer to severe influenza A infection and bars labelled 2 refer to mild influenza A infection. A detailed description of this map can be found at http://www.genego.com/map_707.php. **(c) Pathway diagram for the role of APC in cell cycle regulation.** Red bars indicate up-regulation and blue bars indicate down-regulation. Bars labelled 1 refer to severe influenza A infection and bars labelled 2 refer to mild influenza A infection. A detailed description of this map can be found at http://www.genego.com/map_472.php. **(d) Pathway diagram for role of ATM/ATR regulation of G1/S checkpoint in DNA damage.** Red bars indicate up-regulation and blue bars indicate down-regulation. Bars labelled 1 refer to severe influenza A infection and bars labelled 2 refer to mild influenza A infection. A detailed description of this map can be found at http://www.genego.com/map_426.php. **(e) Leukocyte response to severe infection.** Leukocyte response on day 1 in subjects with severe viral pneumonia and bacterial pneumonia. Data is based on subjects from Severe group (n = 4), Bacterial group (n = 6) and healthy volunteers (n = 18).
(TIF)

Table S1 **Validation of representative genes by polymerase chain reaction (PCR).**
(DOC)
