# Supplementary figures and images for: Aberrant Cell Cycle and Apoptotic Changes Characterise Severe Influenza A Infection – A Meta-Analysis of Genomic Signatures in Circulating Leukocytes
Source: PLoS One. 2011 Mar 8;6(3):e17186. doi: 10.1371/journal.pone.0017186 (PMC3050844; doi:10.1371/journal.pone.0017186)

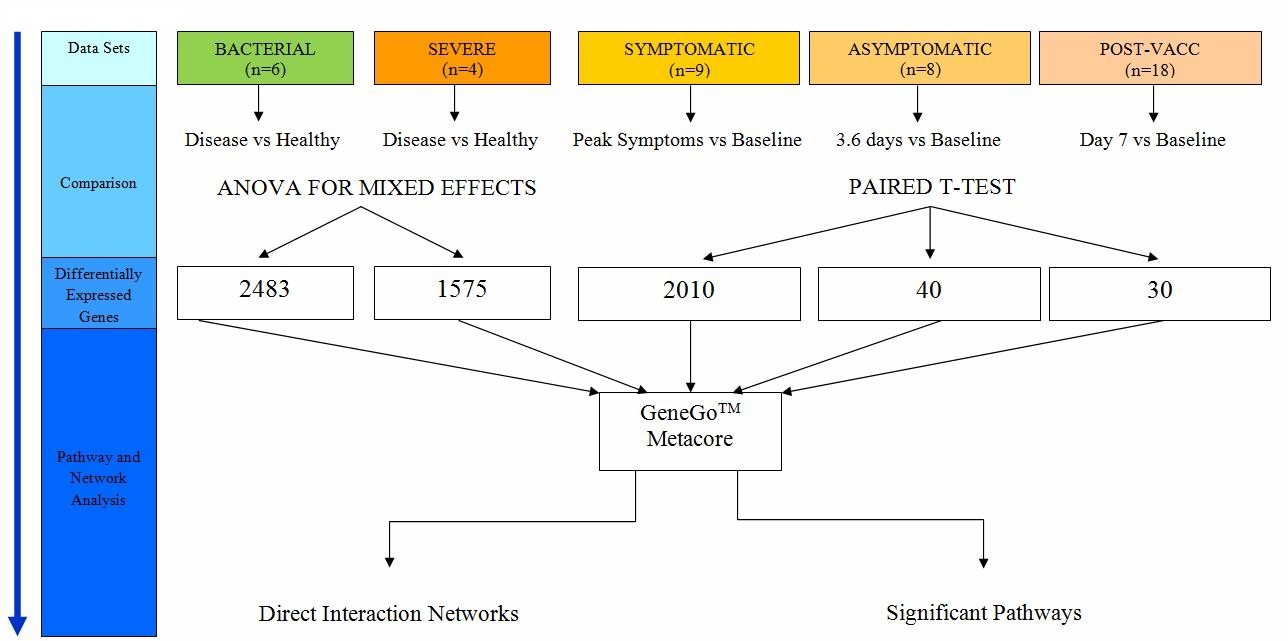

Supplement: Figure S1 — Schematic representation of study design and bioinformatic workflow. (TIF) [file pone.0017186.s001.tif]

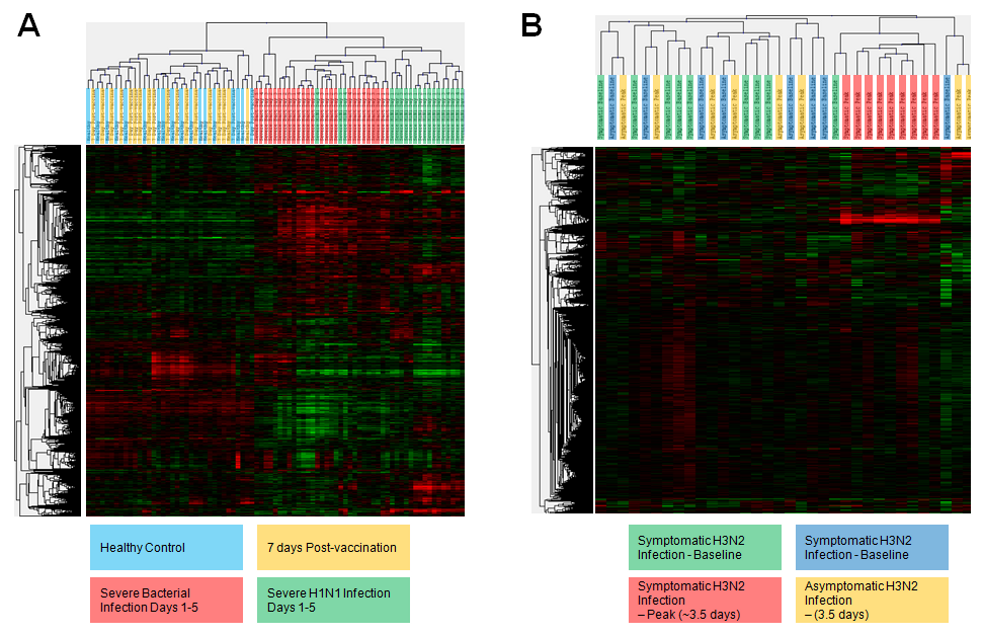

Supplement: Figure S2 — Hierarchical clustering of global gene expression using average linkage and centred correlation. (A) Heatmap of data sets assayed on the Illumina Platform. (B) Heatmap of data sets assayed on the Affymetrix platform. (TIF) [file pone.0017186.s002.tif]

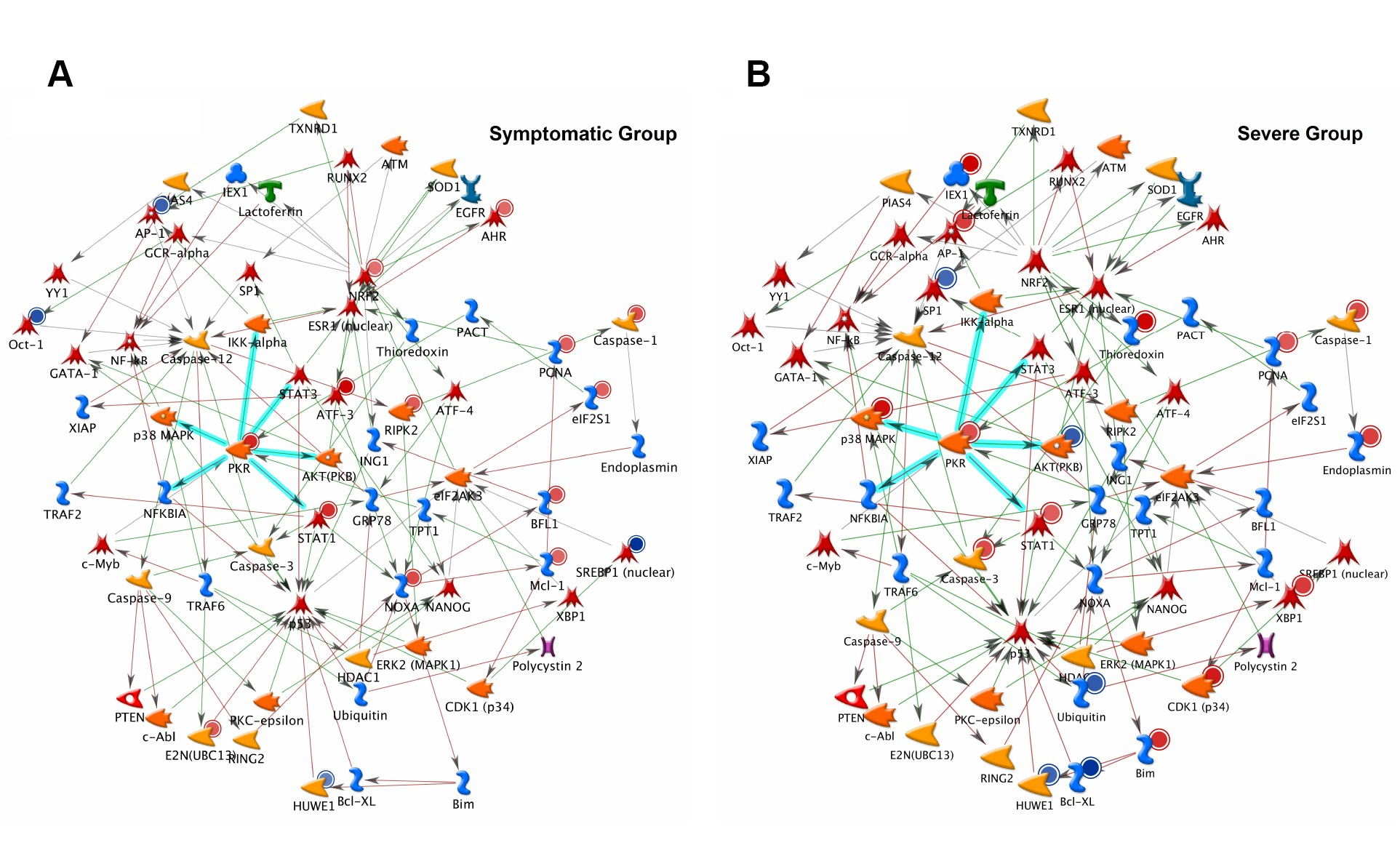

Supplement: Figure S3 — PKR-dependent apoptosis in mild (A) and severe (B) influenza A infection. Pale blue lines indicate direct interaction with PKR. Coloured circles above individual genes indicate up (red) or down (blue) regulation. (TIF) [file pone.0017186.s003.tif]

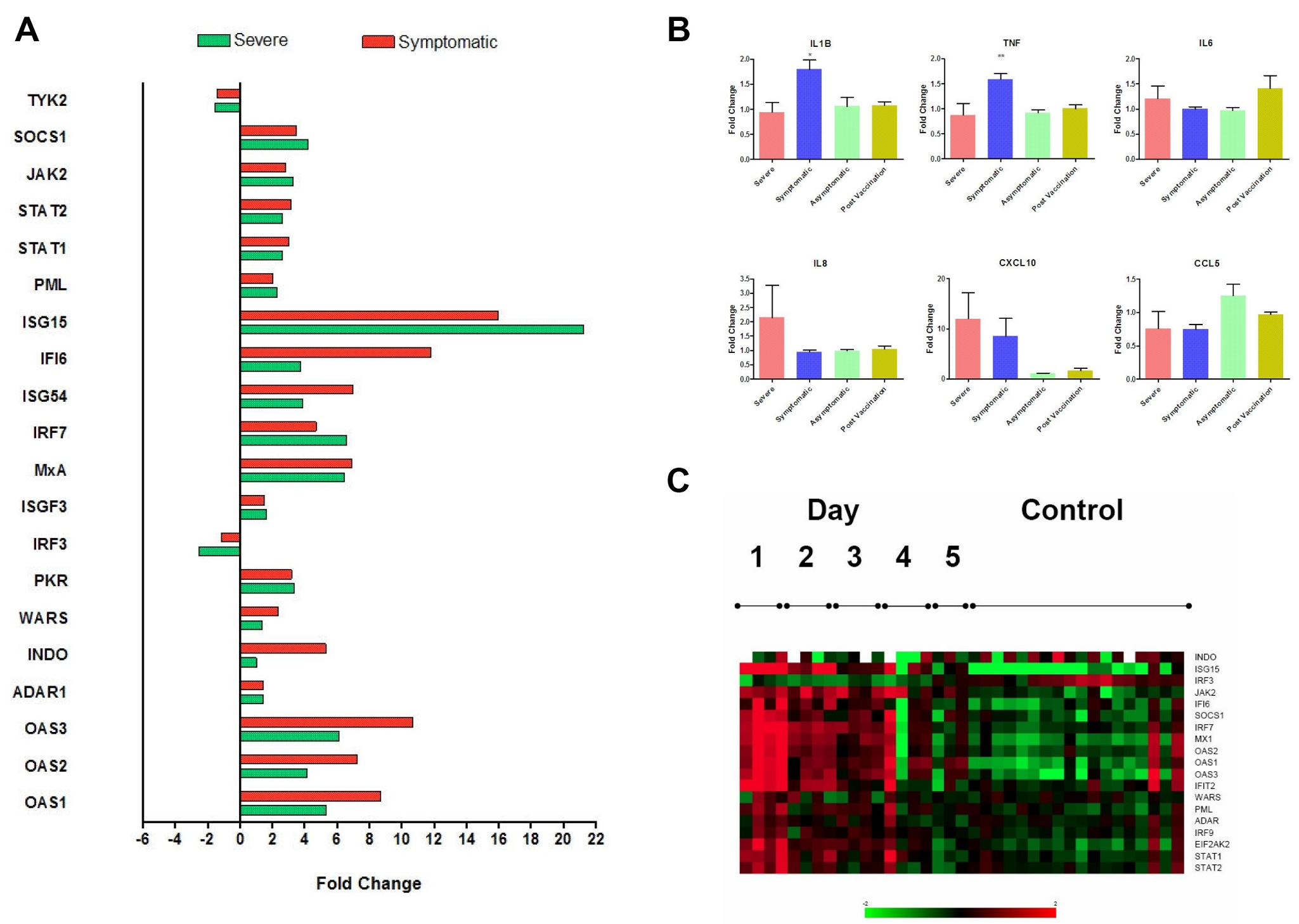

Supplement: Figure S4 — Interferon and Inflammatory response in mild and severe influenza A infection. (a) Expression level of interferon related genes in mild and severe influenza A infection. Day 1 samples are shown for the severe group. (b) Expression level of inflammatory cytokine genes. Day 1 samples are shown for the severe group. (c) Heatmap of interferon related genes during recovery in severe influenza A infection. (TIF) [file pone.0017186.s004.tif]

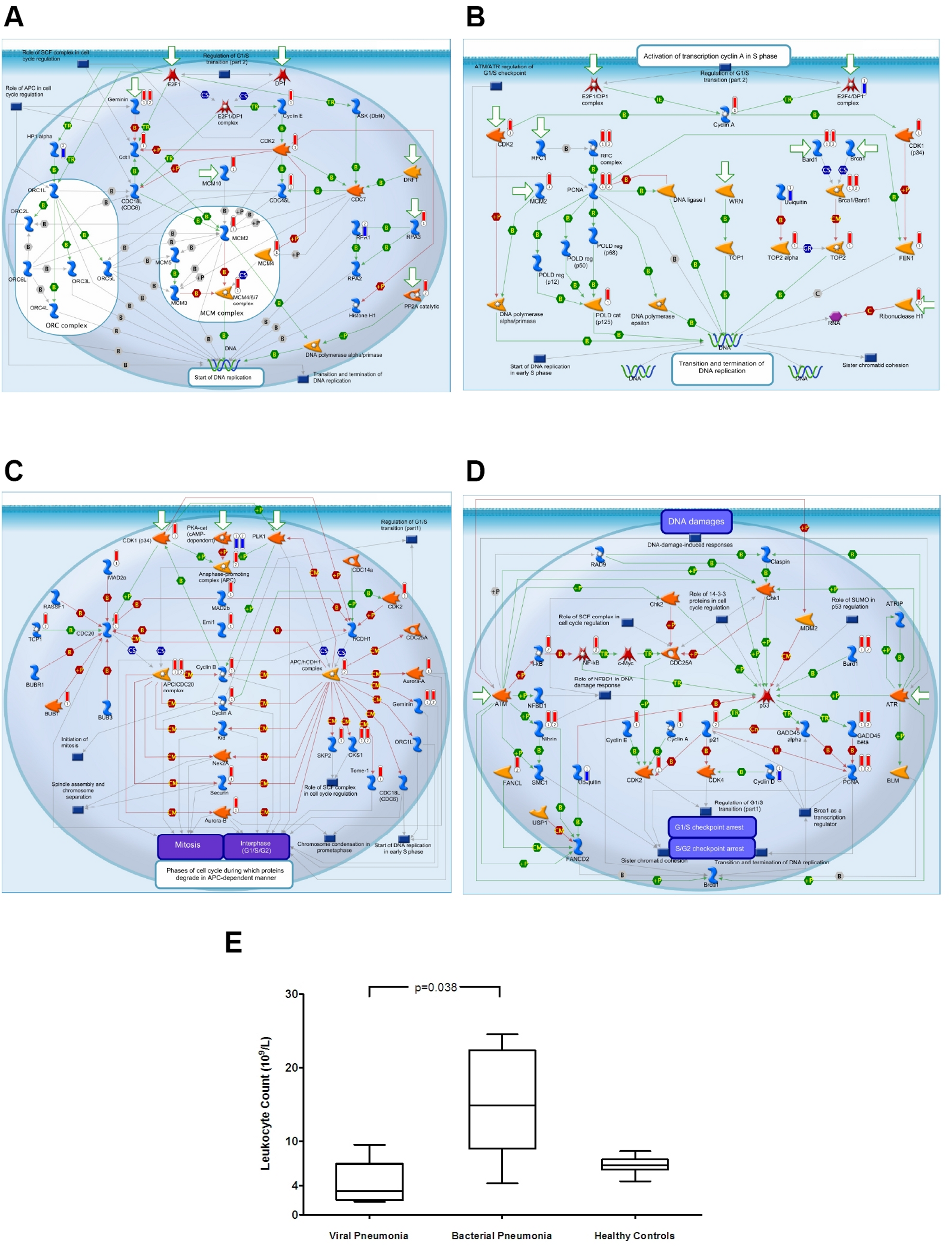

Supplement: Figure S5 — DNA replication and Cell cycle pathways and leukocyte response in severe influenza A infection. (a) Pathway diagram for start of DNA replication in early S phase. Red bars indicate up-regulation and blue bars indicate down-regulation. Bars labelled 1 refer to severe influenza A infection and bars labelled 2 refer to mild influenza A infection. A detailed description of this map can be found at http://www.genego.com/map_705.php. (b) Pathway diagram for transition and termination of DNA replication. Red bars indicate up-regulation and blue bars indicate down-regulation. Bars labelled 1 refer to severe influenza A infection and bars labelled 2 refer to mild influenza A infection. A detailed description of this map can be found at http://www.genego.com/map_707.php. (c) Pathway diagram for the role of APC in cell cycle regulation. Red bars indicate up-regulation and blue bars indicate down-regulation. Bars labelled 1 refer to severe influenza A infection and bars labelled 2 refer to mild influenza A infection. A detailed description of this map can be found at http://www.genego.com/map_472.php. (d) Pathway diagram for role of ATM/ATR regulation of G1/S checkpoint in DNA damage. Red bars indicate up-regulation and blue bars indicate down-regulation. Bars labelled 1 refer to severe influenza A infection and bars labelled 2 refer to mild influenza A infection. A detailed description of this map can be found at http://www.genego.com/map_426.php. (e) Leukocyte response to severe infection. Leukocyte response on day 1 in subjects with severe viral pneumonia and bacterial pneumonia. Data is based on subjects from Severe group (n = 4), Bacterial group (n = 6) and healthy volunteers (n = 18). (TIF) [file pone.0017186.s005.tif]
