# Supplementary material for: Aberrant Cell Cycle and Apoptotic Changes Characterise Severe Influenza A Infection – A Meta-Analysis of Genomic Signatures in Circulating Leukocytes
Source: PLoS One. 2011 Mar 8;6(3):e17186. doi: 10.1371/journal.pone.0017186 (PMC3050844; doi:10.1371/journal.pone.0017186)
Supplement: Table S1 — Validation of representative genes by polymerase chain reaction (PCR). (DOC) [file pone.0017186.s006.doc]

Table S1. Validation of representative genes by polymerase chain reaction (PCR)

| **Gene Name** | **Biological Function** | **Pearson’s Correlation (R^2^)** | ***p*-value** |
| --- | --- | --- | --- |
|  |  |  |  |
| CDC20 | Cell cycle | 0.771606 | < 0.001079 |
| CCNB2 | Cell cycle | 0.825381 | < 0.000545 |
| CASP1 | Apoptosis | 0.674890 | <0.005685 |
| TNFSF10 | Apoptosis | 0.701794 | <0.002424 |
| MX1 | Immune Response | 0.838372 | <0.000458 |

The correlation of gene-expression intensity between by PCR and microarray was assessed using Pearson correlation (R^2^)
